# Supplementary material for: Tumor Infiltrating Lymphocytes in Multi-National Cohorts of Ductal Carcinoma In Situ (DCIS) of Breast
Source: Cancers (Basel). 2022 Aug 13;14(16):3916. doi: 10.3390/cancers14163916 (PMC9406008; doi:10.3390/cancers14163916)
Supplement: Supplementary file 1 [file cancers-14-03916-s001.zip › cancers-1737515-supplementary.pdf]

Supplementary Table S1. Patient characteristics for each DCIS cohort						
	Italian (N=47)	Mayo (N=67)	Oxford (N=69)	Singapore (N=73)	Total (N=256)	p value
**Age**						0.004
N-Miss	0	1	1	1	3	
<=50	8 (17.0%)	7 (10.6%)	20 (29.4%)	25 (34.7%)	60 (23.7%)	
>50	39 (83.0%)	59 (89.4%)	48 (70.6%)	47 (65.3%)	193 (76.3%)	
**Size(mm)**						0.089
N-Miss	3	6	40	1	50	
<=20	29 (65.9%)	45 (73.8%)	19 (65.5%)	38 (52.8%)	131 (63.6%)	
>20	15 (34.1%)	16 (26.2%)	10 (34.5%)	34 (47.2%)	75 (36.4%)	
**grade**						0.215
N-Miss	0	4	8	1	13	
Low	4 (8.5%)	10 (15.9%)	8 (13.1%)	10 (13.9%)	32 (13.2%)	
Intermediate	25 (53.2%)	24 (38.1%)	18 (29.5%)	23 (31.9%)	90 (37.0%)	
High	18 (38.3%)	29 (46.0%)	35 (57.4%)	39 (54.2%)	121 (49.8%)	
**HR**						0.006
N-Miss	0	28	50	8	86	
Positive	40 (85.1%)	30 (76.9%)	10 (52.6%)	57 (87.7%)	137 (80.6%)	
Negative	7 (14.9%)	9 (23.1%)	9 (47.4%)	8 (12.3%)	33 (19.4%)	
**Recurrence**						0.014
N	41 (87.2%)	51 (76.1%)	42 (60.9%)	55 (75.3%)	189 (73.8%)	
Y	6 (12.8%)	16 (23.9%)	27 (39.1%)	18 (24.7%)	67 (26.2%)	
**Ancestry**						
N-Miss	3	1	69	0	73	
Asian	9 (20.5%)	8 (12.1%)	0	73 (100.0%)	90 (49.2%)	
European	35 (79.5%)	58 (87.9%)	0	0 (0.0%)	93 (50.8%)	
**Follow up Time (Years)**						< 0.001
N-Miss	1	2	0	3	6	
All	1 - 12.5 (median = 8.2)	1 – 18 (median = 10)	0.03 - 17.1 (median =7.9)	0.9 - 13.7 (median = 5)	0.03 – 18 (median = 6)	
Non-Recurrent Patients	1 - 12.5 (median = 9.4)	5– 18 (median = 10)	0.03 - 17.1 (median =8.8)	0.9 – 6.2 (median = 5)	0.03 – 18 (median = 7.2)	
Recurrent Patients	1.8 – 10.5 (median = 3.9)	1 – 14(median = 5.5)	0.8 - 15.2 (median =2.8)	1 - 13.7 (median = 3.4)	0.8 – 15.2 (median = 4)	

Supplementary Table S2. Patient characteristics and association with TILs assessments for each DCIS cohort

	Italian (N=47)	Mayo (N=67)	Oxford (N=69)	Singapore (N=73)	Total (N=256)	p value
**Lymphocyte**						0.421
0-5%	21 (44.7%)	28 (41.8%)	29 (42.0%)	23 (31.5%)	101 (39.5%)	
>5%	26 (55.3%)	39 (58.2%)	40 (58.0%)	50 (68.5%)	155 (60.5%)	
**Touching TILs**						0.058
0	44 (93.6%)	59 (88.1%)	54 (78.3%)	57 (78.1%)	214 (83.6%)	
>0	3 (6.4%)	8 (11.9%)	15 (21.7%)	16 (21.9%)	42 (16.4%)	
**Circumferential TILs**						0.022
No	33 (70.2%)	53 (79.1%)	53 (76.8%)	42 (57.5%)	181 (70.7%)	
Yes	14 (29.8%)	14 (20.9%)	16 (23.2%)	31 (42.5%)	75 (29.3%)	
**Hotspot**						< 0.001
No	37 (78.7%)	48 (71.6%)	42 (60.9%)	33 (45.2%)	160 (62.5%)	
dense	10 (21.3%)	19 (28.4%)	27 (39.1%)	40 (54.8%)	96 (37.5%)	

Supplementary Fig S1

Stromal TILs

A

varname	cohort	HR.5yr	p_val.5yr
TILs	singapore	0.54	0.31
TILs	mayo	0.71	0.62
TILs	italien	2.01	0.55
TILs	oxford	0.46	0.08
TILs	all	0.61	0.11

B

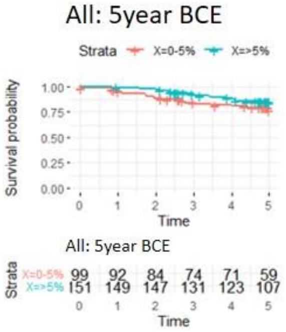

C

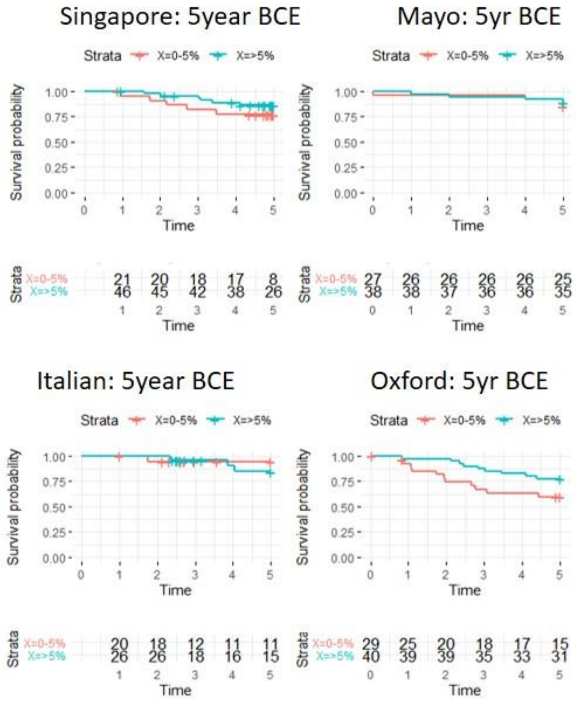

Figure S1. Correlation analysis of tumor-infiltrating lymphocytes (TILs) with clinical outcome for each cohort. (A) The hazard ratio and 95% confidence intervals associated with TILs and 5year recurrence, (B) Kaplan-Meier (KM) plots for 5year recurrence-free survival for TILs for all co-horts, combined, (C) Kaplan-Meier (KM) plots for 5-year recurrence-free survival for TILs for each cohort.

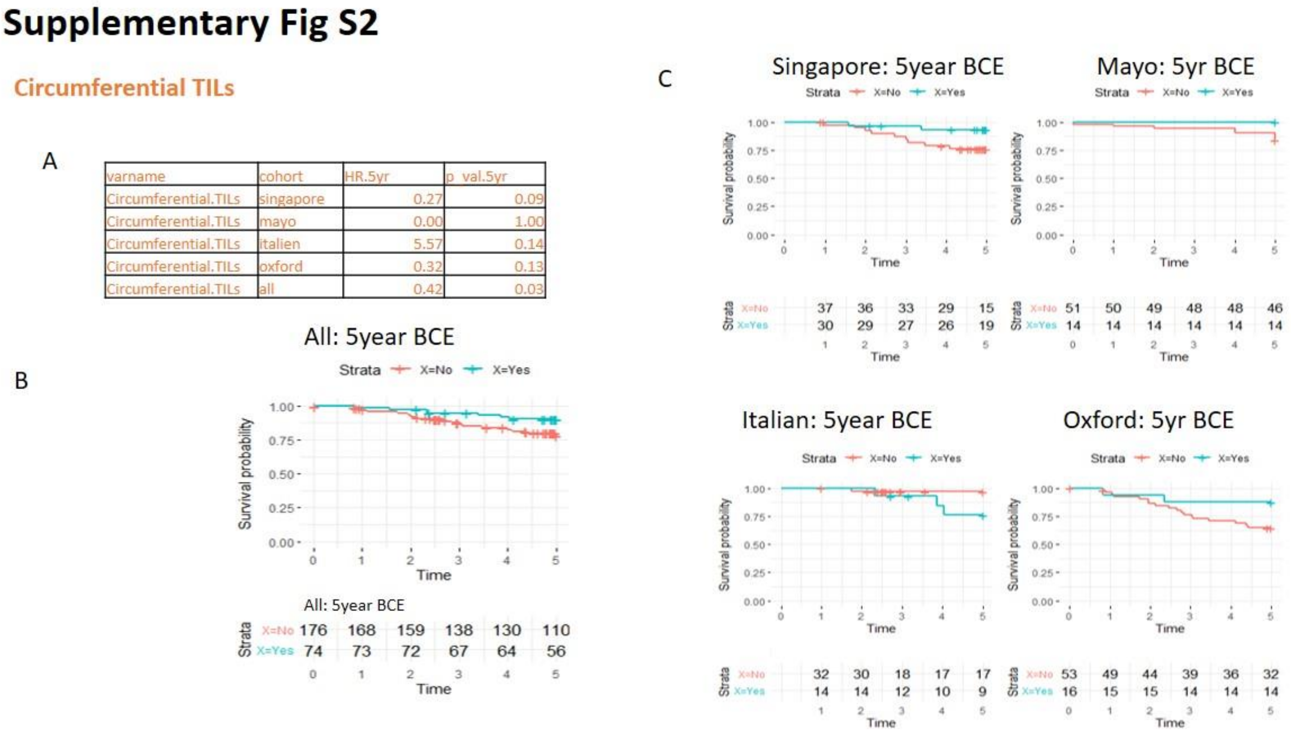

varname	cohort	HR.5yr	p_val.5yr
Circumferential.TILs	singapore	0.27	0.09
Circumferential.TILs	mayo	0.00	1.00
Circumferential.TILs	italien	5.57	0.14
Circumferential.TILs	oxford	0.32	0.13
Circumferential.TILs	all	0.42	0.03

Figure S2. Correlation analysis of touching tumor-infiltrating lymphocytes (touch-ing TILs) with clinical outcome for each cohort. (A) The hazard ratio and 95% confidence inter-vals associated with TILs and 5year recurrence, (B) Kaplan-Meier (KM) plots for 5year recur-rence-free survival for TILs for all cohorts, combined, (C) Kaplan-Meier (KM) plots for 5-year recurrence-free survival for TILs for each cohort.

Supplementary Fig S3

Hotspot TILs

A

varname	cohort	HR.5yr	p_val.5yr
Hotspot	singapore	0.65	0.48
Hotspot	mayo	1.60	0.52
Hotspot	italien	1.04	0.97
Hotspot	oxford	0.72	0.49
Hotspot	all	0.93	0.83

B

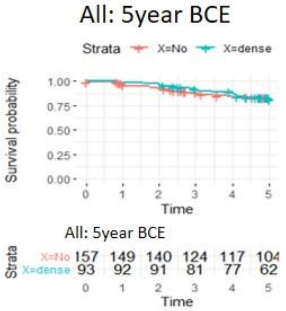

C

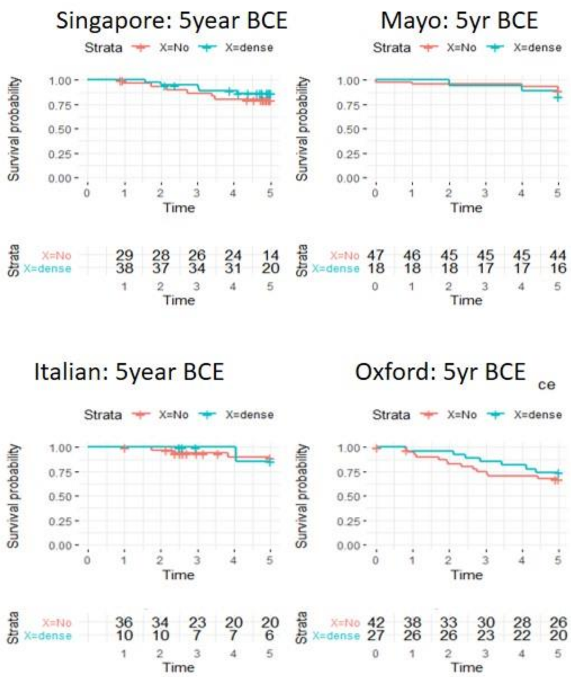

Figure S3. Correlation analysis of circumferen-tial tumor-infiltrating lymphocytes (TILs) with clinical outcome for each cohort. (A) The hazard ratio and 95% confidence intervals associated with TILs and 5year recurrence, (B) Kaplan-Meier (KM) plots for 5year recurrence-free survival for TILs for all cohorts, combined, (C) Kaplan-Meier (KM) plots for 5-year recurrence-free survival for TILs for each cohort.

Supplementary Fig S4

Touching TILs

A

varname	cohort	HR.5yr	p_val.5yr
Touching.TILs	singapore	0.78	0.75
Touching.TILs	mayo	0.00	1.00
Touching.TILs	italien	0.00	1.00
Touching.TILs	oxford	0.61	0.42
Touching.TILs	all	0.63	0.33

B

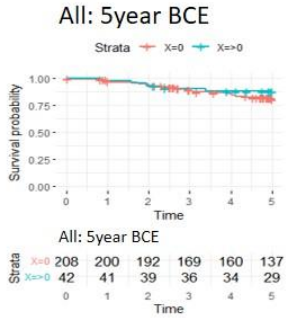

C

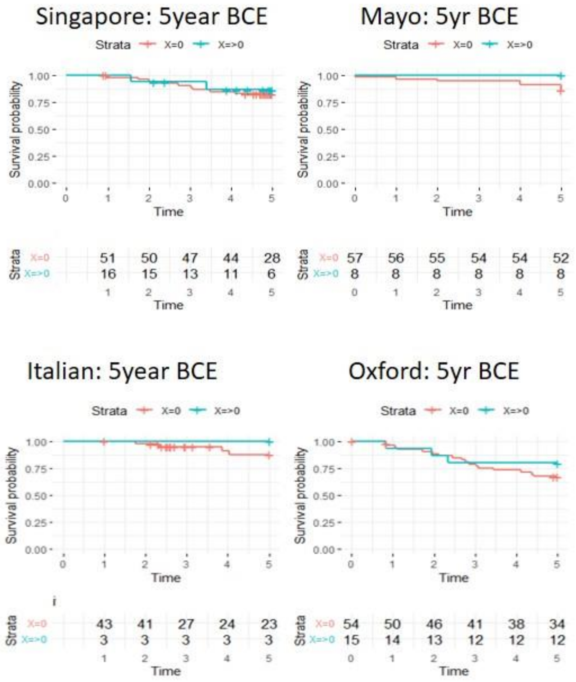

Figure S4. Correlation analysis of hotspot tumor-infiltrating lymphocytes (TILs) with clinical outcome for each cohort. (A) The hazard ratio and 95% confidence intervals associated with TILs and 5year recurrence, (B) Kaplan-Meier (KM) plots for 5year recurrence-free survival for TILs for all co-horts, combined, (C) Kaplan-Meier (KM) plots for 5-year recurrence-free survival for TILs for each cohort.

Supplementary Fig S5

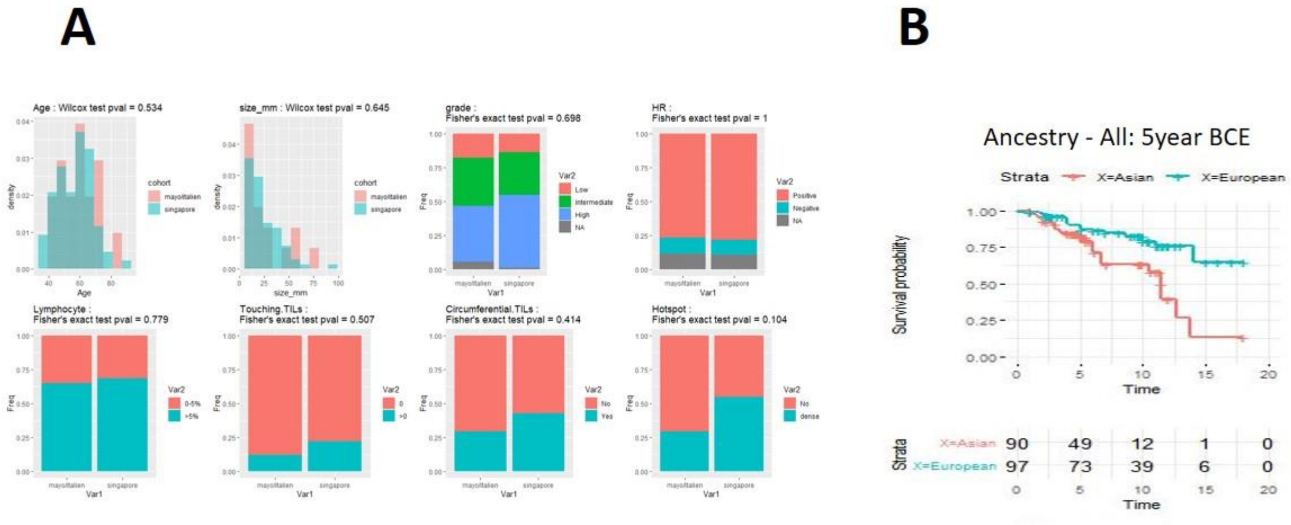

Figure S5. Analysis of clinico-pathological variables, tumor-infiltrating lympho-cytes (TILs) assessment and 5year recurrence risk between Asian and European cohorts. (A) Cor-relation of clinico-pathological variables and tumor-infiltrating lymphocytes (TILs) and (B) KM plot of 5 year recurrence for the Asian and European cohorts.
